# Atypical Manifestation of Adult Polycystic Kidney Disease in an Elderly Individual

**DOI:** 10.7759/cureus.55072

**Published:** 2024-02-27

**Authors:** Oxana Ushakova, Keyvan Ravakhah

**Affiliations:** 1 Internal Medicine, MetroHealth Medical Center, Cleveland, USA; 2 Internal Medicine, Mount Carmel Medical Center, Columbus, USA; 3 Internal Medicine, St. Vincent Charity Medical Center, Cleveland, USA

**Keywords:** oldest patient with adpkd, huge cystic kidney, radiology finding, asymptomatic polycystic kidney disease, asymmetric adpkd

## Abstract

Autosomal dominant polycystic kidney disease (ADPKD) is a rare genetic disease. Diagnosis of ADPKD is usually made by the number of renal cysts on the ultrasound for each age category. There are two types of ADPKD, and the patients with the second type have later onset of symptoms, with slower disease progression than in the first type. These patients are typically at risk of recurrent urinary tract infections, hemorrhage and rupture of cysts, end-stage renal disease, calculi, liver/pancreatic cysts, and brain aneurysm development.

## Introduction

Autosomal dominant polycystic kidney disease (ADPKD) affects approximately 1 in every 500 to 2,500 individuals. [1] Today, our understanding of this condition has deepened, with several gene mutations identified as culprits, including PKD1, PKD2, GANAB, and DNAJB11 [2,3].

The hallmark of ADPKD is the formation of cysts originating from renal tubules, gradually accumulating fluid filtered through the glomerulus. Over time, these cysts grow in size, posing a myriad of clinical challenges. Severity can vary significantly, with truncating mutations in the PKD1 gene often associated with more severe manifestations. Additionally, the interplay of other genetic factors, such as the angiotensin-1-converting enzyme (ACE) gene, cystic fibrosis transmembrane conductance regulator gene, and the tuberous sclerosis complex-2 gene, further influences disease progression [4].

The implications of ADPKD extend beyond the kidneys, with liver cysts affecting over 90% of patients, and approximately 20% of individuals over 60 years of age experiencing brain aneurysms. Recurrent urinary tract infections (UTIs), hypertension, renal calculi, hemorrhage, insufficiency, and proteinuria are common complications, often culminating in end-stage renal disease (ESRD) [1,5,6]. The anticipated lifespan typically falls between 50 and 70 years, with variations based on the specific subtype of ADPKD.

A review of recent literature revealed a few case reports of polycystic kidney disease, mostly manifesting as bilateral or symmetrical cysts with sizes ranging from 5 to 10 cm or less. Asymmetric or unilateral presentations are rare, and these atypical presentations are more frequently observed in children [7-10].

Management strategies for ADPKD aim to alleviate symptoms, slow disease progression, and improve overall quality of life. This often involves the use of ACE inhibitors to enhance renal blood flow and reduce proteinuria, as well as vasopressin receptor antagonists such as tolvaptan which slows cyst growth. In cases where ESRD ensues, options such as hemodialysis or renal transplant become necessary avenues for intervention [11].

Our patient, the oldest documented case to date, showcases an unprecedented asymmetrical and massive cystic burden. Despite the advanced age and the staggering size of the cysts, the patient, whose ADPKD was an accidental finding on computed tomography (CT), was managed without requiring hemodialysis.

## Case presentation

**Figure 1:**
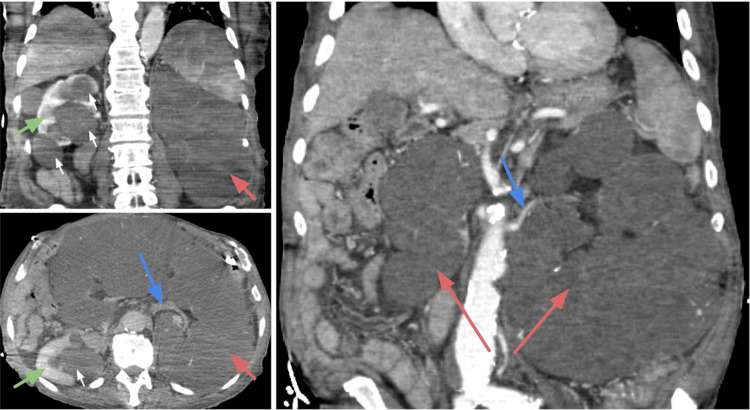
Abdominal CT scan with intravenous contrast. Red arrow: left kidney with cystic changes; blue arrow: left renal artery; green arrow: right kidney; white small arrows: cysts.

A 95-year-old man with a past medical history of adult polycystic kidney disease, hypertension, prostate cancer, sinus bradycardia, and a recent admission due to an uncomplicated UTI two weeks prior was admitted to the hospital with worsening mental status and multiple falls from his bed in a nursing home. A trauma workup was performed in the emergency department, and an abdominal CT scan with intravenous contrast revealed a massive cystic replacement of the left kidney measuring 26.7 x 13.8 x 18.7 cm, crossing the midline and displacing bowel loops (Figure 1). The right kidney also had multiple large cortical and parapelvic cysts, although it was significantly smaller. These findings were consistent with the previous abdominal CT scan conducted 10 years ago. There was only one small 2.8 cm cyst in the liver. No cerebral traumas, hemorrhage, or aneurysms were observed on CT angiography of the head. His family denied any history of UTIs, apart from the recent one. According to the chart records, he was able to produce a normal amount of urine daily. The patient’s blood work results were as follows: hemoglobin of 10.6 g/dL, leukocytes of 2.8 x 10^9^/L, platelets of 37 K/µL, creatinine of 1.07 mg/dL, blood urea nitrogen of 27 mg/dL, and an estimated glomerular filtration rate (eGFR) of 64 mL/minute/1.73m^2^.

Upon admission, the patient was lethargic and oriented only to himself. According to his family, he had a normal mental status, was alert, and fully oriented previously. On the physical examination, he had a normal constitution, a distended abdomen, and a large, uneven mass was palpable. The patient was repeatedly treated for the UTI. During the week of hospitalization, his mental status slowly improved, and he was discharged back to the nursing home in a stable condition.

## Discussion

The primary abnormality driving the formation of cysts in polycystic kidney disease is attributed to dysfunctions in cilia-mediated signaling processes. Polycystic kidney disease is theorized to emerge from deficiencies in the primary cilium, a hair-like structure present on the surface of most cells in the body, firmly anchored within the cell body by the basal body. Renal primary cilia are prevalent on nephron cells. The exact mechanisms through which primary cilium defects contribute to cyst development remain uncertain. One hypothesis suggests disruption in signaling pathways regulated by the primary cilium, such as those involving intracellular calcium, Hedgehog, Wnt/β-catenin, cyclic adenosine monophosphate, or planar cell polarity [11].

ADPKD typically progresses to ESRD, and by the age of 60 years, 50% of patients require dialysis. Treatment approaches depend on the risk of progression, which is determined by factors such as age, eGFR, and kidney size. The strategy revolves around disease progression, prevention, and symptomatic management.

The initial management of ADPKD involves sodium restriction and controlling blood pressure using an ACE inhibitor or an angiotensin receptor blocker to improve renal blood flow and reduce proteinuria. It is recommended that those with an eGFR greater than 30 mL/minute/1.73 m^2^ increase their daily fluid intake to over 3 L, as this is believed to suppress vasopressin production [12-14]. For patients at high risk of rapid progression, tolvaptan, an antagonist of the vasopressin V2 receptor, is offered as a treatment option. This medication is believed to slow cyst growth. The extensive two-phase TEMPO study (Table 1) provides strong evidence of tolvaptan’s efficacy in slowing the progression of ADPKD. In TEMPO 3:4 [15], the authors compared the treated and placebo groups, whereas in TEMPO 4:4 [16], both groups received treatment during the study (with a total of 871 participants). The time interval between the two studies ranged from 13 to 829 days, with a mean of 81 days (Table 1).

**Table 1:** A summary of TEMPO studies for the treatment of ADPKD with tolvaptan. Early-treated: TEMPO 3:4 Tolvaptan group, n = 557; delay-treated: TEMPO 3:4 Placebo group, n = 314. TKV: total kidney volume; eGFR: estimated glomerular filtration rate; ADPKD: autosomal dominant polycystic kidney disease

	TEMPO 3:4 [10]		P-value	TEMPO 4:4 [11]		P-value
Groups	Tolvaptan group	Placebo group	N/A	Early-treated	Delay-treated	N/A
Duration	36 months			24 months		
TKV (mL), median (normal range for men = 137 ± 28 mL)	1,498 (977) mL	1,468 (751) mL	0.6	1,706 (1,199) mL	1,835 (1,162) mL	0.05
TKV, % change from TEMPO 3:4	N/A			15.8% (17.0%)	23.8% (16.9%)	<0.0001
eGFR, [mean (SD)] (normal range >90 mL/minute/1.73m^2^)	82.2 (20.6) mL/minute/1.73m^2^	83.5 (22.6) mL/minute/1.73m^2^	0.59	72.3 (24.5) mL/minute/1.73m^2^	70.4 (25.0) mL/minute/1.73m^2^	0.38
eGFR, change from TEMPO 3:4 [mean (SD)]	N/A			−8.1 (−10.3) mL/minute/1.73m^2^	−10.6 (−13.71) mL/minute/1.73m^2^	0.003

Another treatment option is kidney transplantation, which is reserved for patients who do not respond to medical treatment and develop ESRD [17].

## Conclusions

Our patient is the oldest individual with ADPKD documented in the literature. This patient has a positive influence on the life expectancy of this patient population. We believe that his second kidney, which has fewer cysts, managed to compensate and maintain a normal eGFR for his age. Furthermore, the uneven enlargement of the right kidney and its size posed a challenge in diagnosing polycystic kidney disease in this man. However, an experienced radiologist, through meticulous evaluation and a thorough search for renal arteries, was able to identify the source of the mass.
